# Small Luggage for a Long Journey: Transfer of Vesicle-Enclosed Small RNA in Interspecies Communication

**DOI:** 10.3389/fmicb.2017.00377

**Published:** 2017-03-16

**Authors:** Fabio A. Lefebvre, Eric Lécuyer

**Affiliations:** ^1^Institut de Recherches Cliniques de Montréal (IRCM), RNA Biology DepartmentMontreal, QC, Canada; ^2^Département de Biochimie, Université de MontréalMontreal, QC, Canada; ^3^Divison of Experimental Medicine, McGill UniversityMontreal, QC, Canada

**Keywords:** extracellular vesicles, exosomes, miRNA, small RNA, communication, argonaute, gene silencing

## Abstract

In the evolutionary arms race, symbionts have evolved means to modulate each other's physiology, oftentimes through the dissemination of biological signals. Beyond small molecules and proteins, recent evidence shows that small RNA molecules are transferred between organisms and transmit functional RNA interference signals across biological species. However, the mechanisms through which specific RNAs involved in cross-species communication are sorted for secretion and protected from degradation in the environment remain largely enigmatic. Over the last decade, extracellular vesicles have emerged as prominent vehicles of biological signals. They can stabilize specific RNA transcripts in biological fluids and selectively deliver them to recipient cells. Here, we review examples of small RNA transfers between plants and bacterial, fungal, and animal symbionts. We also discuss the transmission of RNA interference signals from intestinal cells to populations of the gut microbiota, along with its roles in intestinal homeostasis. We suggest that extracellular vesicles may contribute to inter-species crosstalk mediated by small RNA. We review the mechanisms of RNA sorting to extracellular vesicles and evaluate their relevance in cross-species communication by discussing conservation, stability, stoichiometry, and co-occurrence of vesicles with alternative communication vehicles.

## Introduction

The “one gene, one enzyme” paradigm has long dominated our understanding of molecular biology. Although peptides are key effectors of cell physiology, strictly protein-centrist portraits of life have encountered early criticism and been deemed reductive since the 1950s (Mc, 1950). In the post-genomic era, it has become increasingly clear that the bulk of eukaryotic genomes—loci previously dubbed “junk DNA” or “dark matter”—undergo pervasive transcription, yielding thousands of non-coding (nc)RNAs, many of which are conserved and tissue-specific (Clark et al., 2011; Coffey et al., 2011; Derrien et al., 2012; Jalali et al., 2016). In particular, small non-coding (s)RNAs transcribed from intergenic, intronic, and repeated regions can exert RNA interference (RNAi) by guiding Argonaute ribonucleases (RNAses) to specific complementary targets (Hammond et al., 2001). The pivotal role of sRNA in a broad range of biological contexts is well-established: estimates suggest that up to 60% of mammalian mRNA is subjected to RNAi by sRNA (Lewis et al., 2005).

Symbiotic relationships favor the intricate proximity of multiple species in biological niches. To sustain the evolutionary arms race, symbionts have evolved means to influence each other via secreted signals. In line with the “one gene, one enzyme” paradigm, communication across species was long taught to strictly involve peptides and small metabolites. Over the last decade, however, reports of communication across species via transfers of RNA silencing signals have surged in diverse biological niches, prompting a re-evaluation of cross-species communication (Knip et al., 2014; Weiberg et al., 2015).

Naked RNA transcripts are rapidly degraded in the human systemic circulation (Tsui et al., 2002) but extracellular vesicles (EVs), ribonucleoprotein complexes (RNPs) and lipoproteins can stabilize transcripts and protect them from RNAse degradation (Valadi et al., 2007; Hunter et al., 2008; Lasser et al., 2011; Lefebvre et al., 2016). Microarray and deep-sequencing approaches have revealed that repertoires of secreted sRNA don't mirror cellular populations, suggesting the involvement of selective sorting mechanisms (Valadi et al., 2007; Hunter et al., 2008; Lasser et al., 2011; Lefebvre et al., 2016). Various proteins of the RNAi machinery have been found in mammalian EVs and can perform cell-independent sRNA maturation (Melo et al., 2014). In addition, RNA silencing activity can be transferred across tissues, with emerging implications in early development (da Silveira et al., 2015; Sharma et al., 2016), cancer biology (Melo et al., 2014; Dror et al., 2016), immunology (Montecalvo et al., 2012), regenerative medicine (Hergenreider et al., 2012), and gene therapy (Mizrak et al., 2013). Recent reports show that EVs enable inter-organismal and long-range transfers of functional RNA and proteins in *C. elegans* (Wang et al., 2014) and in mammals (Viss et al., 2003), suggesting that EVs may contribute to cross-species transfer of RNA silencing activity.

Here, we survey the properties of eukaryotic sRNA and review emerging evidence of inter-organismal RNAi across species and kingdoms, including crosstalk between plants, bacterial, fungal, and metazoan pathogens and host-microbiota interactions in the gut. We discuss the mechanisms of RNA sorting to mammalian EVs for secretion. We suggest that sRNA-loaded EVs may contribute to cross-species RNAi activity. To put this hypothesis in perspective, we discuss contrasting evidence challenging the efficiency of EV-mediated sRNA transfer in light of deficient stability and stoichiometry.

## Overview of gene silencing by sRNA

**Figure 1:**
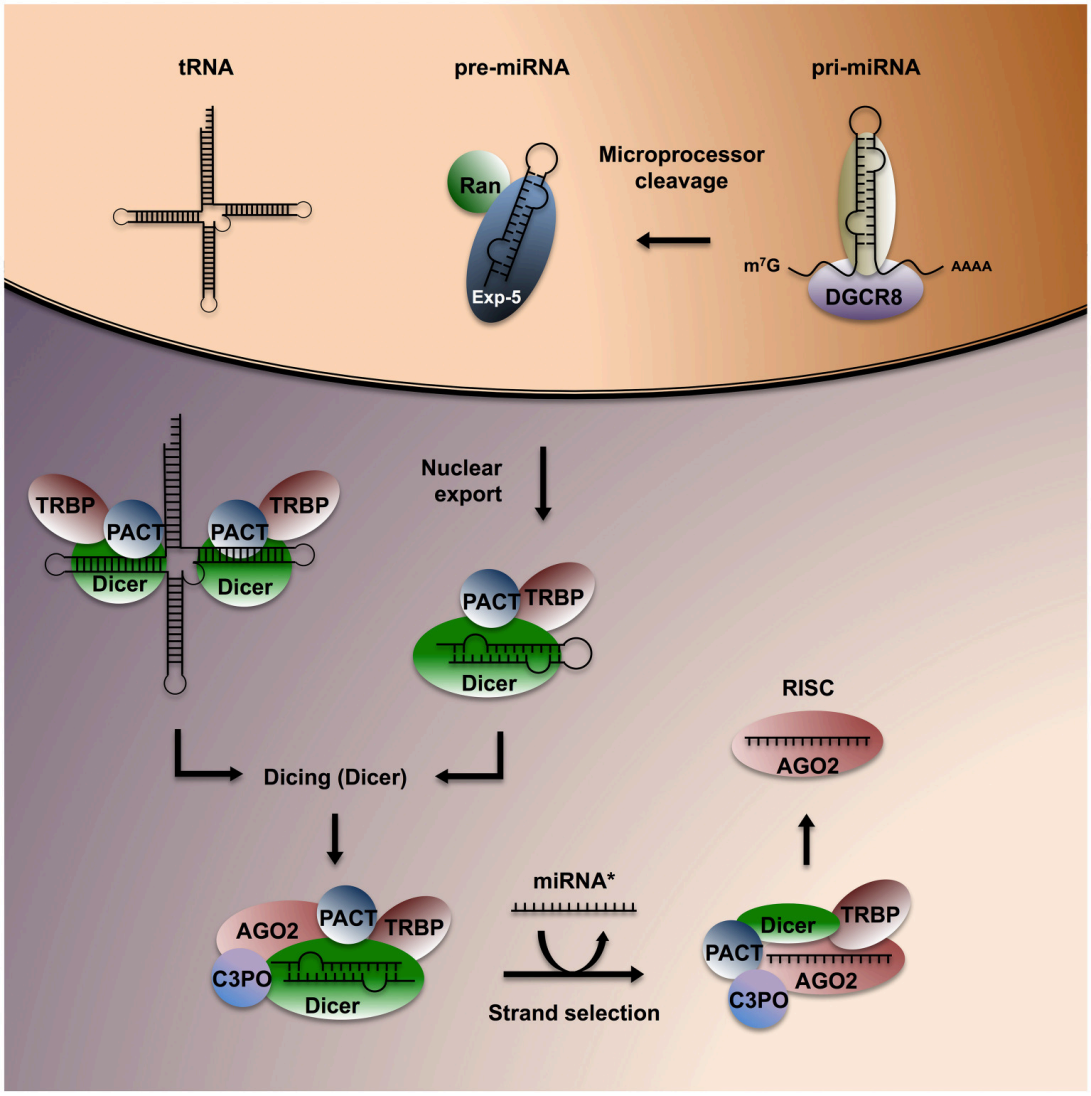
**Overview of sRNA biogenesis in mammals**. Pri-miRNA is cleaved into pre-miRNA by the microprocessor complex, consisting of two nuclear proteins, Drosha and its cofactor DGCR8. Pre-miRNA is exported to the cytoplasm through Exportin-5 (Exp-5), then bound and processed into short dsRNA sequences of ~22 nt by the RBP Dicer and its associated factors TRBP and PACT. Structured ncRNA encompassing stretches of paired nucleotides such as tRNAs can also be recognized and processed as Dicer substrates. Dicer recruits AGO2 and its cleavage yields two single-stranded RNA sequences, called the leading strand and the guide strand (or miRNA^*^). The leading strand is actively repositioned in the complex and loaded onto AGO2 to form a RISC, which can exert RNA silencing.

Small RNAs have three defining features: (1) they are short (21–31 nucleotides), (2) don't encode peptides, and (3) can associate with RNAses of the Argonaute family (AGO) to modulate gene expression by targeting complementary transcripts. sRNA can impact gene expression through at least four distinct mechanisms: (1) AGO-dependent cleavage of target RNA, (2) destabilization of target mRNA through polyA tail shortening, (3) translational inhibition via polysomal protein interactions, and (4) transcriptional silencing through chromatin modifications (Volpe et al., 2002; Ghildiyal and Zamore, 2009; Rissland and Lai, 2011). The field of RNAi truly emerged in the late 1990s, after the discovery that double-stranded (ds) RNA can specifically silence complementary transcripts in *C. elegans* (Fire et al., 1998). An expanding repertoire of sRNA classes has since surfaced, including microRNA (miRNA), endogenous small interfering RNA (endo-siRNA), and Piwi-interacting RNA (piRNA). In addition, infrastructural non-coding RNA, such as transfer RNA (tRNA) and vaultRNA (vtRNA) can be processed and recognized by the RNAi machinery to silence diverse mRNA targets (Persson et al., 2009; Sharma et al., 2016). The protein machinery involved in sRNA nuclear export, maturation and RNAi is highly conserved and was likely present in the last common ancestor of eukaryotes (Shabalina and Koonin, 2008), emphasizing the potential functionality of RNAi in cross-species communication. The biogenesis of sRNA in mammals has been clarified over the last decade (Lee et al., 2004; Forstemann et al., 2005; Yeom et al., 2006) and reviewed in detail elsewhere (Mattick and Makunin, 2005; Collins and Cheng, 2006; Ghildiyal and Zamore, 2009; Rissland and Lai, 2011; Figure 1).

Most miRNAs are transcribed from intergenic or intronic regions, with a few examples derived from exons of protein-coding genes. Hairpins found on primary (pri)-miRNA transcripts (≈1,000 nt) bind to the nuclear microprocessor complex consisting of the RNAse III enzyme Drosha and the RBP DGCR8/Pasha. The microprocessor cleaves pri-miRNAs into precursor (pre)-miRNAs (≈70 nt), which undergo nuclear export via Exportin-5 (Yi et al., 2003). Pre-miRNA and long dsRNA sequences are recognized by Dicer and subsequently cleaved (“diced”) to generate sRNA duplexes (≈22 nt). Recent evidence suggests that structured dsRNA regions of tRNA and vtRNA can also be recognized and processed by Dicer, yielding small sequences similar to a mature miRNA (Persson et al., 2009). Argonaute 2 (AGO2) is recruited to the complex by the Dicer-binding protein TRBP, enabling the transfer of the leading (or guide) RNA strand to the PAZ domain of AGO2. TRBP, PACT and C3PO are involved in leading strand selection and re-positioning (Noland et al., 2011; Noland and Doudna, 2013). They also clear the remaining complementary single-stranded RNA copy, the passenger strand, or miRNA^*^, which is typically targeted for degradation (Liu et al., 2009). The resulting minimal RNA-induced silencing complex (RISC) consists of AGO2 bound to the mature miRNA.

## RNAi in crosstalk between plants, bacteria, fungi, insects and nematodes

Plants rely on RNAi for various endogenous processes, including defense against viral parasites. The genome of *Arabidopsis thaliana* encodes over 10 different AGO proteins, presumably reflecting the emergence of new functions (Zhang et al., 2015). In 1990, Napoli et al. reported an unexpected block in anthocyanin biosynthesis upon the introduction of a chimeric chalcone synthase construct in petunia (Napoli et al., 1990). The resulting crop exhibited white and patterned flowers presenting pale non-clonal sectors on a pigmented *wild type* (WT) background. The mechanism involved was deemed unclear at the time and only became apparent after the realization that dsRNA expression leads to gene silencing (Fire et al., 1998). More recently, engineering of plant RNAi has been described as an effective and “eco-friendly” method to modulate crop phenotypes in the aim of increasing productivity (Younis et al., 2014). In particular, host gene silencing–hairpin RNAi (HGS-hpRNAi), wherein a transgenic sRNA-encoding hairpin is expressed in plants has emerged over the last 15 years to impact pathogen resistance (Viss et al., 2003). HGS-hpRNAi is reportedly effective against diverse pathogens, including bacteria (e.g., *Agrobacterium*), fungi (e.g., *Fusarium*), insects (e.g., *Helicoverpa*) and nematodes (e.g., *Meloidogyne*; Table 1). However, the mechanisms through which plant-encoded dsRNA and/or sRNA molecules are transferred to symbiotic neighbors remain largely unclear and could involve EVs, RNPs and/or lipoproteins.

**Table 1:** **Evidence of RNAi activity transfers from plants to bacteria, fungi and metazoans**.

**Donor plant**	**Recipient pathogen**	**Targets**	**Outcome**	**References**
*Arabidopsis thaliana* (Thale cress)	*Agrobacterium tumefaciens* (Bacteria)	*iaaM, ipt*	Resistance to crown gall disease	Escobar et al., 2001
*Malus* genus (Apple)	*Agrobacterium tumefaciens* (Bacteria)	*iaaM, ipt, iaaH*	Resistance to crown gall disease	Viss et al., 2003
*Juglans regia* (Walnut)	*Agrobacterium tumefaciens* (Bacteria)	*iaaM, ipt*	Resistance to crown gall disease	Escobar et al., 2002
*Hordeum vulgare* (Barley)	*Fusarium graminearum* (Fungus)	*CYP51*	Inhibition of fungal growth	Koch et al., 2013
*Musa paradisiaca* (Banana)	*Fusarium oxysporum* (Fungus)	*Velvet, FTG1*	Effective resistance to *F. oxysporum*	Ghag et al., 2014
*Nicotiana* genus (Tobacco)	*Phytophtora capsici* (Fungus)	*PcAvr3a1*	Infection of resistant tobacco	Vega-Arreguin et al., 2014
*Medicago truncatula* (Barrelclover)	*Glomus intraradices* (Fungus)	*MST2*	Impaired mycorrhiza formation	Helber et al., 2011
*Malus* genus (Apple)	*Venturia inequalis* (Fungus)	*THN*	Light brown phenotype	Fitzgerald et al., 2004
*Solanum tuberosum* (Potato)	*Meloidogyne* sp. (Nematode)	*Mc16D10L*	Egg count reduction	Dinh et al., 2014
*Glycine max* (Soybean)	*Meloidogyne incognita* (Nematode)	*TP, MSP*	Reduction of *incognita* gall count	Ibrahim et al., 2011
*Zea mays* (Maize)	*Diabrotica virgifera* (Insect)	*v-ATPaseA/E β-tubulin*	Increased larval mortality	Baum et al., 2007
*Nicotiana benthamiana*	*Myzus persicae* (Insect)	*Rack1 MpC002*	Reduced aphid fecundity	Pitino et al., 2011
*Arabidopsis thaliana* (Thale cress)	*Helicoverpa armigera* (Insect)	*CYP6AE14*	Larval growth retardation	Mao et al., 2007
*Nicotiana rustica* (Tobacco)	*Helicoverpa armigera* (Insect)	*EcR*	Improvement of pest resistance	Zhu et al., 2012
*Nicotiana rustica* (Tobacco)	*Bemisia tabaci* (Insect)	*v-ATPseA*	Improvement of pest resistance	Thakur et al., 2014
*Medicago sativa* (Lucerne)	*Acyrthosiphon pisum* (Insect)	*COO2*	Lethality of *A. pisum*	Mutti et al., 2006
*Oryza sativa* (Rice)	*Nilaparvata lugens* (Insect)	*NIHT1, Nlcar Nltry*	No phenotype reported	Zha et al., 2011

The soil bacterium *Agrobacterium tumefaciens* causes crown gall disease through disruptions of host's auxin and cytokinin biosynthesis, leading to the formation of tumor in various species of *Eudicotidae* flowering plants (Hoekema et al., 1983). The pathogenesis of *Agrobacterium* crown gall disease is well- characterized and involves the bacterial Tumor-inducing (Ti) plasmid (McCullen and Binns, 2006). Ti ssDNA is trafficked from the bacterium to the host plant via a conjugation pilus (McCullen and Binns, 2006), which is a prevalent vehicle of nucleic acid exchange among bacteria. Whether the bacterial conjugation pilus can enable host-encoded sRNA populations to enter *Agrobacterium* remains unclear. Ti is integrated in the host genome by recombination and encodes opine synthesis oncogenes (*iaaM* and *ipt*). Escobar et al. reported resistance to crown gall tumorigenesis in transgenic *Arabidopsis* expressing self-complementary constructions designed to initiate HGS-hpRNAi against *iaaM* and *ipt* (Escobar et al., 2001). This finding was later confirmed in apple (*Malus pumila*) and walnut (*Juglans regia*) trees (Escobar et al., 2002; Viss et al., 2003).

**Figure 2:**
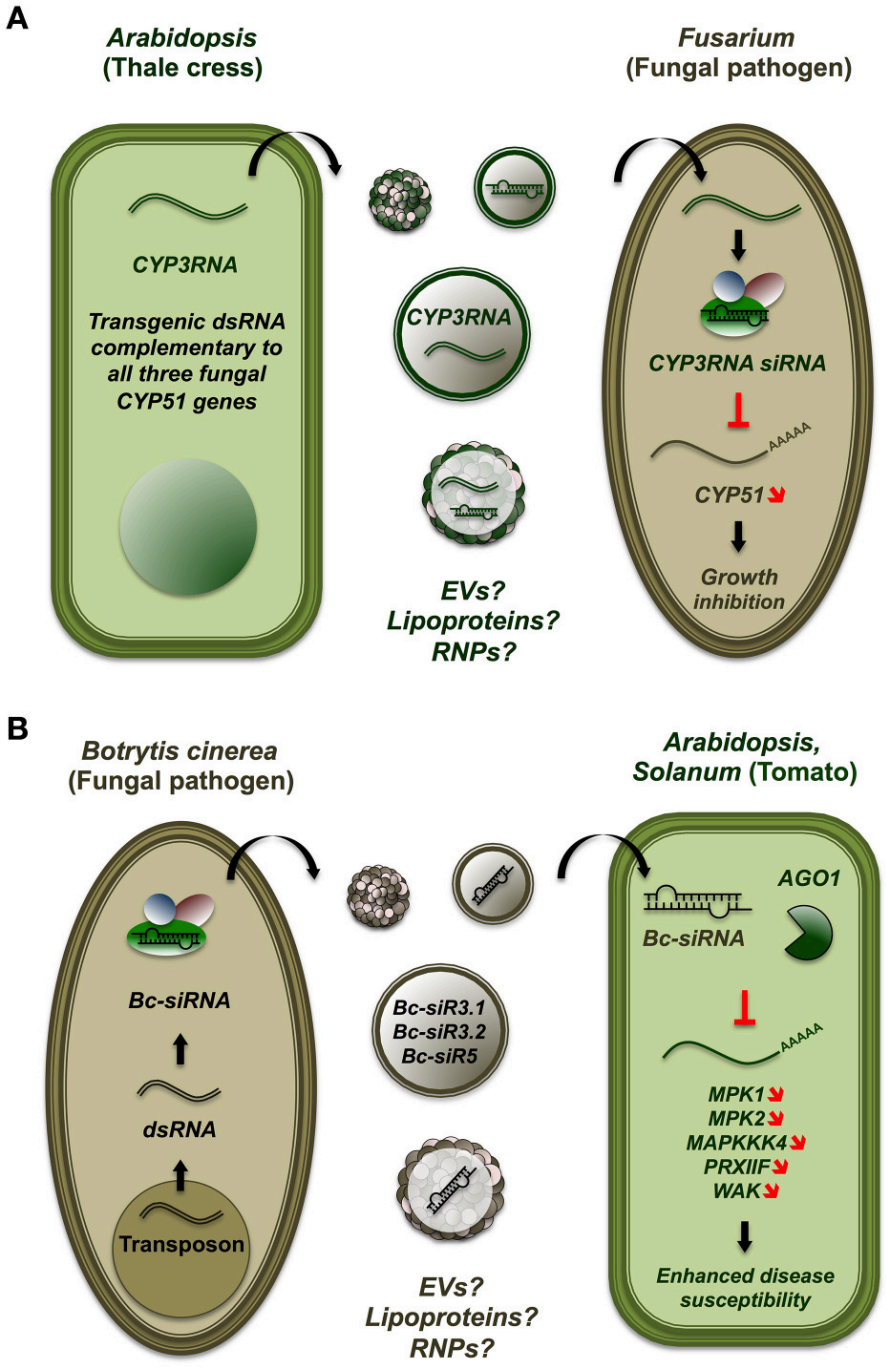
**Host-induced hairpin RNA-mediated silencing confers resistance to the fungal pathogen ***Fusarium***. (A)** Host-induced hairpin RNA-mediated silencing enables plant to resist to the fungal pathogen *Fusarium*. In *Arabidopsis*, expression of a dsRNA construct complementary to fungal *CYP51* transcripts can immunize transgenic plants to the pathogenic ascomycete *Fusarium graminearum* by inhibiting fungal growth (Koch et al., 2013). The vehicles through which transgenic dsRNA and/or sRNA is transferred are unknown and possibly include plant EVs, secreted RNPs and/or lipoproteins. **(B)** *Botrytis cinerea* sRNA populations hijack *Arabidopsis* RNAi pathways to suppress plant immunity. Populations of sRNA derived from a *B. cinerea* retrotransposon are shuttled to infected *Arabidopsis* and *Solanum lycopersicum*. In plants, fungal sRNA are loaded onto AGO1 and direct the silencing of diverse proteins, including Mitogen-activated kinases, which impact the host's immune response (Weiberg et al., 2013). The vehicles through which dsRNA and/or sRNA are transferred from fungus to plant are unknown and possibly include fungal EVs, secreted RNPs and/or lipoproteins.

Ascomycete pathogens of the *Fusarium* genus release mycotoxins and cause “root rot” and *Fusarium* head blight pathologies, leading annually to severe loss in cereal crop productions. Koch et al. showed that *A. thaliana* and *Hordeum vulgare* (barley) plants expressing dsRNA targeting the fungal gene *CYP51* were completely immune to *Fusarium graminearum* (Koch et al., 2013; Figure 2). In transgenic plants, fungal growth was strongly restricted (≥99%) and only present in the vicinity of inoculation sites. The authors reported increased sporulation and altered morphology of *Fusarium* exposed to transgenic plants, consistent with compromised levels of cytochrome P450 lanosterol C-14α-demethylase, the enzyme encoded by *CYP51*. Similarly, Ghag et al. achieved effective resistance to *Fusarium oxysporum cubense* in transgenic bananas (*Musa paradisiaca*) expressing hairpins to target two vital fungal genes, *velvet* and *Fusarium transcription factor 1* (Ghag et al., 2014). Additional demonstrations of HGS-hpRNAi from plants to fungi have involved the mutualistic mycorrihzal genus *Glomus*. Helber et al. characterized the *Monosaccharide Transporter2* (*MST2*) gene in *Glomus* species and provided evidence of its requirement for mycorrhiza formation through a HGS-hpRNAi loss-of-function model (Helber et al., 2011). A similar approach was used by Vega-Arreguin et al. to demonstrate the role of the fungal gene *Avr3a1* in the hypersensitive response exhibited by diverse species of the genus *Nicotiana* to the pathogenic oomycete *Phytophthora capsici* (Vega-Arreguin et al., 2014).

Nematodes are appealing models to study RNAi and HGS-hpRNAi. In the nematode *C. elegans*, where RNAi was discovered (Fire et al., 1998), sRNA induces a systemic, amplified, and heritable response (Collins and Cheng, 2006). Strong evidence indicates that dsRNA expressed in *E. coli* fed to *C. elegans* can transmit systemic and heritable silencing activity upon ingestion (Liu et al., 2012). In *C. elegans*, the intestinal transmembrane protein SID-1 binds and imports dsRNA (Jose et al., 2009), while SID-2 is associated with cellular export of RNAi signals and required for systemic environmental RNAi (Winston et al., 2007). Parasitic nematodes of the *Meloidogyne* genus are soilborne root pathogens that feed on diverse plants, notably potato (*Solanum tuberosum*) and soybean (*Glycine max*). *Meloidogyne* damages roots and compromises the plant's ability to absorb water and nutrients, affecting crop productivity (Abad et al., 2008). Dinh et al. showed that *Arabidopsis* and potato plants expressing dsRNA constructs that target the nematode gene *16D10L* develop resistance to *Meloidogyne chitwoodi*. Interestingly, RNAi against *16D10L* was transferred to the progeny of worms feeding on transgenic roots, consistent with reports of heritable RNAi in nematodes (Dinh et al., 2014). In addition, Ibrahim et al. assessed the efficiency of HGS-hpRNAi at reducing galls formed by *Meloidogyne incognita* in soybean roots. The authors tested four potential targets in *M. incognita* and reported that HGS-hpRNAi against transcripts encoding Tyrosine Phosphatase (TP) and Mitochondrial Stress-70 Protein Pre-cursor (MSP) were highly efficient at reducing galls in infected plants (Ibrahim et al., 2011).

Like nematodes, several arthropods can internalize dietary dsRNA molecules (Khila and Grbic, 2007). Indeed, orthologs of the *sid-1* gene have been identified in several insects, including *Apis mellifera* (honeybee), *Bombyx mori* (silkworm), and *Tribolium castaneum* (red flour beetle; Gramates, 2006). In light of these findings, Baum et al. provided a general assessment of HGS-hpRNAi usefulness for the control of coleopteran insects. Baum et al. showed that the western corn rootworm *Diabrotica virgifera virgifera* Leconte is sensitive to orally provided dsRNA, exhibiting dramatic suppression of 17 endogenous targets within 24 h of ingestion (Baum et al., 2007). The authors demonstrated the potential of oral dsRNA delivery for insect pest control by determining the lethal dose of sequences targeting diverse protein-coding genes. Among the 290 dsRNA tested, 125 showed significant larval mortality. In a HGS-hpRNAi assay, maize plants expressing dsRNA targeting coleopteran *v-ATPse A* were protected from *Diabrotica* feeding damage. Thakur et al. and Mutti et al. provided additional evidence that expression of dsRNA targeting insect genes can improve crop resistance in various models (Mutti et al., 2006; Thakur et al., 2014). Multiple plants release secondary metabolites or phytochemicals that promote resistance to parasites. For example, cotton plants (*Gossypium* genus) synthesize gossypol, a toxic sesquiterpene compound that detracts most herbivores (Mao et al., 2007). However, the cotton bollworm, *Helicoverpa armigera*, tolerates high concentrations of gossypol. Mao et al. showed that a cytochrome P450 gene, *CYP6AE14*, is induced by gossypol and required for insect tolerance to the compound (Mao et al., 2007). When fed *Arabidposis* or *Nicotiana* plant material expressing a *dsRNA* construct raised against *CYP6AE14*, the sensitivity of *Helicoverpa* larvae to gossypol was markedly increased.

Recent evidence suggests that fungal pathogens can transfer RNAi signals to modulate the immunity of the plants they parasite. Indeed, Weiberg et al. reported that *Botyris cinerea*, the causative agent of gray mold disease, encodes sRNA populations derived from retrotransposons which can silence *Arabidopsis* and *Solanum* genes involved in immunity (Weiberg et al., 2013; Figure 2). In this study, Weiberg et al. generated sRNA sequencing libraries from *B. cinerea* mycelia, conidiospores and total biomass. In parallel, they profiled leaves from *B. cinerea*-infected *Arabidopsis* and tomato plants (*Solanum lycopersicum*). Interestingly, a total of 832 *B. cinerea*-encoded sRNAs were tracked and overrepresented in infected plant extracts, 52 of which mapped to six different fungal long terminal repeat (LTR) retrotransposons. Among predicted *Arabidopsis* and *Solanum* targets, reporter assays confirmed *mitogen activated protein kinases* (*MPK1, MPK2, MAPKKK4*), *peroxiredoxin* (*PRXIIF*), and *cell-wall associated kinase* (*WAK*). Transgenic *Arabidopsis* plants ectopically expressing three *B. cinerea* sRNAs (*Bc-siRNA3.1, Bc-siRNA3.2, Bc-siRNA5*) displayed normal morphology but enhanced disease susceptibility upon pathogen challenge. A consistent phenotype was observed in *mpk1 mpk2* double mutants, suggesting that these factors are involved in host immunity against *B. cinerea*. Immunoprecipitation of AGO1 in *B. cinerea*-infected *Arabidopsis* retrieved *Bc-siRNA3.1, Bc-siRNA3.2 and Bc-siRNA5*. *Arabidopsis* mutants of *Ago1* (*ago1-27*) showed reduced disease susceptibility to *B. cinerea*, whereas the Dicer-like mutant *dcl1-7* displayed enhanced disease phenotype. By contrast, knock out of *B. cinerea dcl* genes depleted sRNA pools and reduced virulence upon *Arabidopsis* and *Solanum* inoculation. Together, these results show that fungal Dicer and plant Ago1 are involved in *Bc-siRNA3.1-, Bc-siRNA3.2-, and Bc-siRNA5*-induced gene silencing in *Arabidopsis*. Weiberg et al. thus unraveled a mechanism whereby fungal sRNA populations hijack the plant host's RNAi machinery to subvert plant immunity and promote disease progression.

## RNAi in crosstalk between intestinal cells and the gut microbiota

With over 100 trillion organisms representing 1,000 species, the gut microbiota plays pivotal roles in human health and disease (Ley et al., 2006; Faith et al., 2013). Inflammatory bowel disease (Halfvarson et al., 2008), diabetes (Patterson et al., 2016), obesity (Tilg et al., 2009; Nehra et al., 2016), diverse malignancies (Louis et al., 2014; 2012), and neurological disorders (Moos et al., 2016) have been linked to disruptions in intestinal homeostasis. Several studies have reported increased parasite susceptibility in mice bearing a conditional *Dicer* deletion in intestinal epithelial cells (*Dicer1*^Δgut^), suggesting that RNAi is involved in mucosal immunity, possibly affecting communication with gut microorganisms.

**Figure 3:**
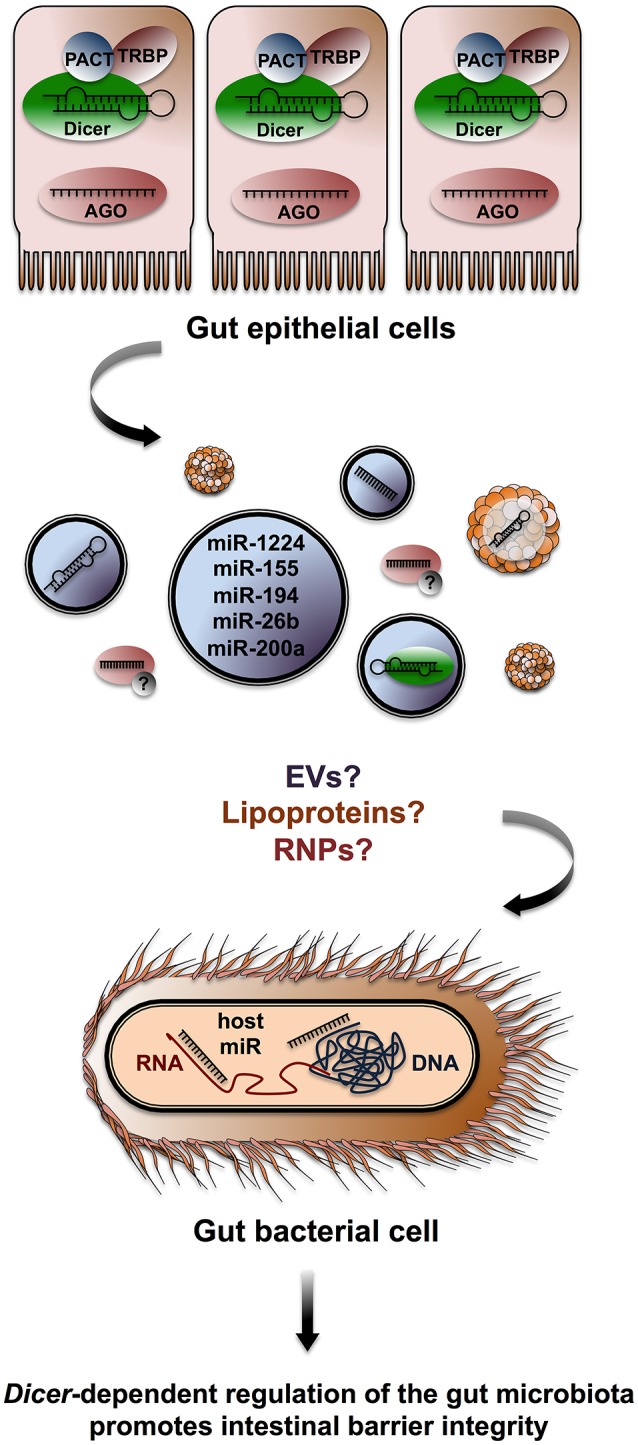
**Host miRNA targets microbiota gene expression**. Gut epithelial cells release miRNAs that can be recovered in murine and human fecal matter. Fecal miRNA populations are likely stabilized through EVs and possibly through lipoproteins or RNPs containing AGO2. Host miRNA enters *E. coli* and *F. nucleatum* where it co-localizes with bacterial nucleic acids and impacts bacterial growth by interacting with nucleic acids (Liu et al., 2016).

Singh et al. identified 16 miRNA transcripts differentially expressed in caecum samples from germ-free and conventionally fed mice. Computational approaches pointed to over 2,000 putative mRNA targets, including factors involved in intestinal barrier function and immune regulation (Singh et al., 2012). The authors found a strong overlap between their list of target mRNA and a previous survey of factors deregulated in the mucosa of *Dicer1*^Δgut^ mice. This observation suggests that the gut microbiota modulates miRNA expression in the host, impacting intestinal barrier integrity. Along with several other studies (Dalmasso et al., 2011; Dai et al., 2015; Runtsch et al., 2015), Singh et al. provides evidence that RNAi modulates crosstalk between gut epithelial cells and the microorganisms that surround them. However, the prevalence and relevance of direct transfers of sRNA molecules from host to gut microbiota remain unclear. In a recent study, Liu et al. contributes to bridge that gap, showing that commensal gut bacteria uptake host miRNAs secreted in feces and that host miRNAs can exert RNAi in *E. coli* and *F. nucleatum* (Liu et al., 2016; Figure 3). Liu et al. (2016) isolated and characterized miRNA populations contained within EVs in human and mice fecal samples. They identified over 180 miRNA in feces, which were differentially distributed in gut luminal content from the distal ileum and colon of mice. They show that intestinal epithelial cells, Paneth cells, and goblet cells all contribute miRNA transcripts that account for fecal populations.

Liu et al. (2016) compared the gut microbiota in fecal matter from *Dicer1*^Δgut^ and control (*Dicer1*^fl/fl^) mice by sequencing the V4 region of rRNA 16S. Several differences were noted: representation of the bacterial phyla Firmicutes and Proteobacteria was notably increased in *Dicer1*^Δgut^ samples. Liu et al. (2016) then submitted seven abundant bacterial RNA sequences from *E. coli* and *F. nucleatum* to a miRBase analysis (Kozomara and Griffiths-Jones, 2014) and identified numerous putative base-pairing events with host miRNA. Synthesized miRNA mimics of hsa-miR-1226-5p promoted the growth of *E. coli*, while hsa-miR-515-5p favored *F. nucleatum in vitro*. Mutated controls preventing base pairing of miRNA mimics to bacterial targets had no impact. Fluorescent Cy3-conjugated miRNA entered *E. coli* and *F. nucleatum*, co-localized with nucleic acids and increased the 16S rRNA/23S rRNA ratio in *F. nucleatum*. In *E. coli*, RNAseP levels were increased by miR-4747-3p while the bacterial transcripts *rutA* and *fucO* levels were decreased by miR-1224-p and miR-623, respectively. Target levels where not affected by mice miRNA mimics bearing mutations in predicted base-pairing nucleotides. Fecal gavage of *Dicer1*^Δgut^ mice with WT samples led to a restoration of WT microbiota populations after 7 days, as determined by 16S rRNA sequencing.

Next, Liu et al. (2016) investigated the phenotype of *Dicer1*^Δgut^ mice and found evidence of reduced MHCII levels in intestinal lymphoid tissue inducer cells. Diverse cytokines were also decreased in the ileum and colon, including LT-β, IFN-γ, and TGF-β. The resistin-like molecules Relm-α and Relm-β, which are critical for maintenance of intestinal barrier integrity, were compromised in the ileum of *Dicer1*^Δgut^ mice, along with Occludin-1, ZO-1, and Claudin-1, -2, and -5, echoing previous reports of *Dicer1*^Δgut^ phenotypes (Braniste et al., 2014). Based on these findings, the authors suspected increased susceptibility to colitis in *Dicer1*^Δgut^ mice, and tested the hypothesis by inducing the disease through oral administration of dextran sulfate sodium. As expected, *Dicer1*^Δgut^ mice exhibited greater body weight loss, colon shortening, and colon infiltration in response to dextran sulfate than WT mice. However, gavage of *Dicer1*^Δgut^ mice with fecal matter from WT mice prior to dextran sulfate treatment alleviated the severity of these phenotypes, suggesting that fecal miRNA can attenuate the colonic alterations seen in *Dicer1*^Δgut^ mice.

Together, these observations strongly suggest that host miRNA can be internalized, exert RNAi, and mediate compositional changes in gut bacterial populations to promote intestinal homeostasis. Although Liu et al. (2016) does not directly demonstrate that host miRNA populations are transferred via EVs, the study shows that sequences enriched in fecal EVs are involved in cross-kingdom RNAi. Interestingly, in mice, EVs have been identified as a communication vehicle between intestinal epithelial cells and the immune system enabling MHCII protein transfers (Van Niel et al., 2003).

## Diverse subpopulations of secreted vesicles contain sRNA

In mammalian cells, the release of membranous vesicles upon exocytosis of vesicular endosomes was first reported in 1983 by Harding et al. (1983). Long dismissed as cellular debris, EVs have emerged over the last decade as key vehicles of biological signals, notably sRNA. Transcripts enriched in EVs include specific mRNA and full-length and fragmented non-coding transcripts, such as ribosomal (r)RNA, long non-coding (lnc)RNA, transfer (t)RNA, vault (vt)RNA, Y RNA, small nuclear (sn)RNA, and small nucleolar (sno)RNA populations (Kalra et al., 2012; Nolte-'t Hoen et al., 2012; Xiao et al., 2012; Li et al., 2013; Lefebvre et al., 2016). Viral transcripts have been identified in EVs of cells infected with Epstein-Barr virus (Pegtel et al., 2010; Nanbo et al., 2013). Although specific miRNAs are overrepresented in EVs, diverse studies indicate that cumulative miRNA abundance is lower in EVs than in cells (Chevillet et al., 2014; Koppers-Lalic et al., 2014).

The EV field have largely focussed on mammalian systems. In humans, EVs have notably been described as promising sources of biomarkers for diverse diseases (Skog et al., 2013). EVs and EV-associated RNA populations have been identified and profiled by RNA-seq in multiple human biological fluids, including blood (Mitchell et al., 2008; Huang et al., 2013), milk (Chen et al., 2014), semen (Vojtech et al., 2014), saliva (Michael et al., 2010), cerebral spinal fluid (Baraniskin et al., 2011), urine (Nilsson et al., 2009), and ascitic fluids (Kahlert and Kalluri, 2013). The release of exosome-like vesicles carrying sRNA populations has also been described in the nematode *Caenorhabditis elegans*, the arthropod *Drosophila melanogaster* (Lefebvre et al., 2016) and the unicellular fungi *Cryptococcus neoformans, Paracoccidioides brasiliensis, Candida albicans*, and *Saccharomyces cerevisae* (Peres da Silva et al., 2015). Similarly, specific populations of sRNA have been defined in protozoans of the *Leishmania* (Lambertz et al., 2015) and *Trypanosoma* (Fernandez-Calero et al., 2015) genera. Outer membrane vesicles released by Gram-negative bacteria, notably *Vibrio cholera*, have been shown to contain specific RNA populations and suggested to function as an RNA delivery system during infection. Release of MVE-associated exosomes in plants has been hypothesized 40 years ago (Halperin and Jensen, 1967) and is consistent with electron microscopy evidence (Tanchak and Fowke, 1987).

“EV” is an umbrella term referring to diverse subpopulations of membrane-enclosed vesicles, often co-purified together in protocols that involve sequential ultracentrifugation of biological fluids (Hill et al., 2013). Exosomes are small EVs (40–120 nm) that originate in endosomes and are released in the extracellular space upon exocytosis of multivesicular endosomes (MVEs). Vesicles shed by the plasma membrane through an actin-dependent abscission are typically larger (50–1,000 nm) and have been called microvesicles, ectosomes, or microparticles (Akers et al., 2013). Apoptotic cells release small vesicles (50–500 nm) in addition to large apoptotic bodies containing organelles (Ihara et al., 1998; Elmore, 2007; 50–5,000 nm). Membrane-enclosed particles with retroviral-like composition and morphology (90–100 nm) have also been identified in cancer cell media (Muster et al., 2003) and in plasma samples of lymphoma patients (Contreras-Galindo et al., 2008). Beyond EVs, sRNA can be stably shuttled in biological fluids in association with lipoproteins and RNP complexes, some containing AGO2 and Nucleophosmin-1 (NPM1). At least two studies suggest that extracellular AGO2 and miRNA are more abundant in soluble complexes than within EVs (Arroyo et al., 2011; Turchinovich et al., 2011). In addition, “tunneling nanotubes” are actin-rich protrusions that can bridge eukaryotic cells and may provide an alternative route for nucleic acid transfers (Belting and Wittrup, 2008). First described in 2004 in cultures of rat pheochromocytoma cells (Rustom et al., 2004), tunneling nanotubes have since been shown to enable transmission of HIV particles between T cells and Jurkat cells (Sowinski et al., 2008).

It is technically challenging to separate subpopulations of EVs. Kowal et al. developed a tailored approach to separate EV subpopulations using continuous density gradients and immuno-isolation (Kowal et al., 2016). Mass spectrometry revealed distinct but partially overlapping protein profiles in human exosomes, microvesicles, and apoptotic bodies. Other studies reported divergent nucleic acid imprints in EV subpopulations (Crescitelli et al., 2013; Lazaro-Ibanez et al., 2014). Crescitelli et al. found that ribosomal RNA are more abundant in apoptotic bodies and in microvesicles than in exosomes, which reportedly contain more RNA than microvesicles. However, Kanada et al. showed that microvesicles could target reporter molecules to recipient cells more efficiently than exosomes, which appeared ineffective at delivering nucleic acids (Kanada et al., 2015).

## Mechanisms of sRNA sorting to EVs

**Figure 4:**
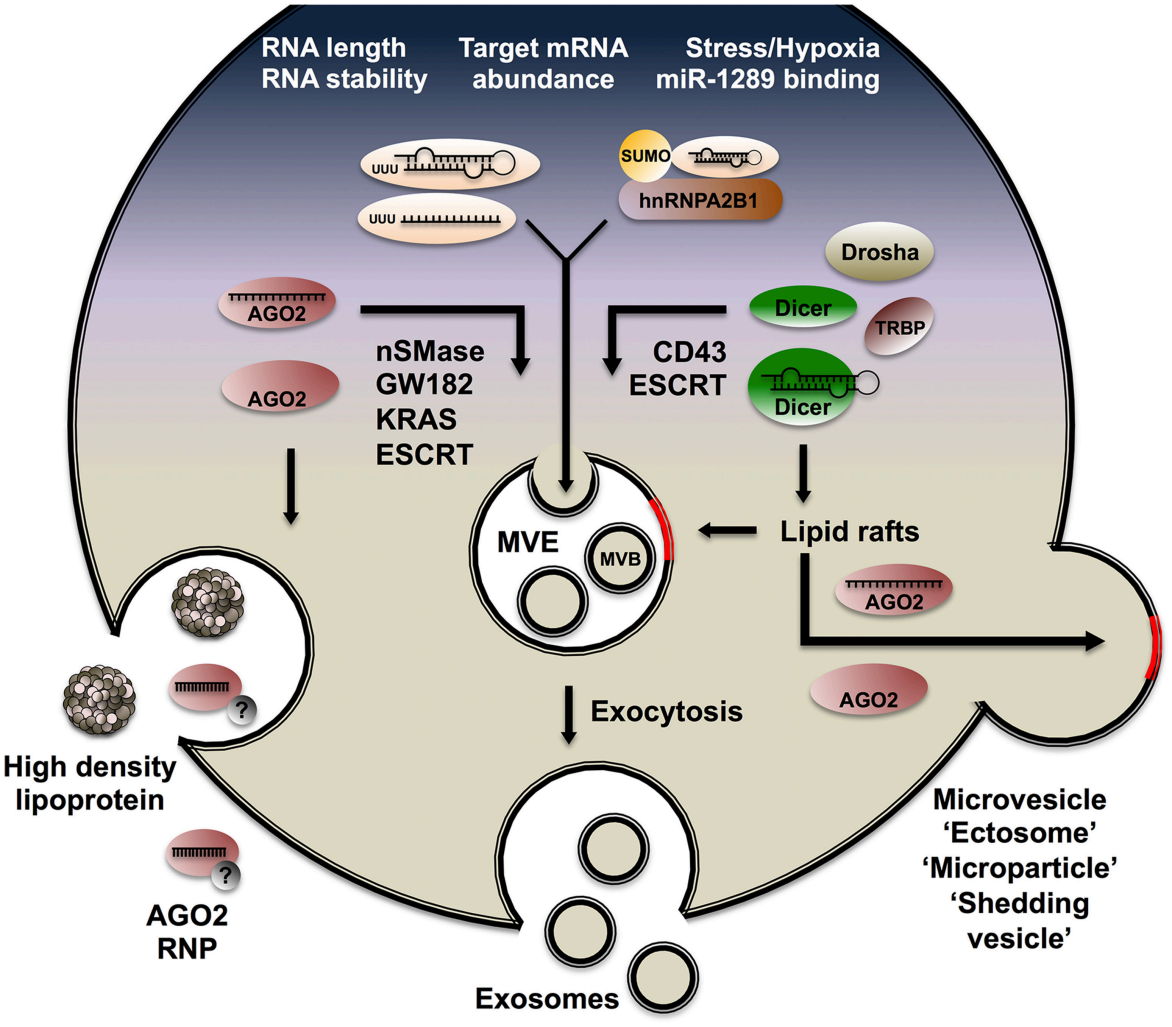
**Mechanisms of sRNA loading to EVs**. Schematic view of a mammalian cell releasing sRNA through lipoproteins and AGO2 RNPs (left), exosomes (center), and membrane-shed microvesicles (right). Properties broadly associated with RNA targeting to EVs are listed on top (white font). Mechanisms, lipid structures and RBPs involved in sorting RNA molecules to exosome and microvesicles are portrayed.

Our understanding of EVs in health and disease has expanded rapidly over the last years and has been frequently reviewed (Stoorvogel et al., 2002; Raposo and Stoorvogel, 2013). In this section, we will focus on the mechanisms involved in sorting sRNA to mammalian EVs (Figure 4). Spatially resolved subcellular targeting of RNA molecules is often mediated by sequence or structure motifs found in the transcript. Called *cis*-acting elements, these sequences specifically interact with *trans*-acting factors, usually RBPs (Martin and Ephrussi, 2009; Cody et al., 2013). At the subcellular level, mRNA localization is a prevalent process with key functional contributions, notably in embryogenesis (MacDonald, 1990; Lecuyer et al., 2007; Cody et al., 2013) and synaptogenesis (Latham et al., 1994; Czaplinski and Singer, 2006; Du et al., 2007). Batagov et al. (2011) extended the rationale of subcellular localization and submitted a list of EV-targeted transcripts inferred from microarray datasets (Skog et al., 2008) to motif search algorithms. Although multiple alignments and position-specific scoring approaches failed to identify shared signatures among EV RNA, the authors found that EV-enriched transcripts display significantly shorter half-lives than cell-retained transcripts. By contrast, Bolukbasi et al. identified a 25 nt motif in the 3′UTR of mRNAs enriched in EVs from glioblastoma and melanoma cell lines (Bolukbasi et al., 2012). Mutagenesis and reporter assays confirmed the functionality of the sequence at targeting mRNA to EVs. Interestingly, the authors found that the motif encompasses the seed region for miR-1289 along with a core CUGCC sequence. Furthermore, altering the levels of miR-1289 was sufficient to modulate artificial target levels in EVs, suggesting a role for miRNA in sorting complementary mRNA to EVs. It should be noted that the studies discussed above used microarray data focussed on mRNA and long non-coding RNA. Other sources have since emphasized the enrichment of shorter sequences in EVs, including specific miRNA (Bellingham et al., 2012). Hung and Leonard showed that RNA length alone strongly modulates targeting efficiency (Hung and Leonard, 2016). They developed a tailored approach based on the MS2-GFP system and confirmed that long sequences (1.5 kb) are poorly loaded in EVs.

EV-associated miRNA repertoires exhibit considerable cell-type specificity and dramatic alterations in these populations have been observed upon cell fate commitment or changes in environmental status and stimuli. Hypoxic conditions have been shown to increase exosomal release and modulate associated miRNA in breast cancer cell lines, notably leading to a strong increase in miR-210 targeting (King et al., 2012). Interleukin treatment also promotes activation-dependent changes in miRNA populations released by macrophages through EVs (Squadrito et al., 2014). The miRNA repertoire of colon cancer cell EVs is profoundly affected by mutations in the transcription factor *KRAS*. Cha et al. profiled the transcriptome of EVs released by cell lines differing only in *KRAS* status and showed that levels of the pro-metastatic miR-100 are decreased in mutant *KRAS* EVs, whereas miR-10b abundance is increased in these samples (Cha et al., 2015).

Unlike EVs released by non-malignant cells, breast cancer exosomes contain the proteins Dicer, TRBP and AGO2, which can perform cell-independent miRNA processing within EVs (Melo et al., 2014). Several studies have identified populations of pre-miRNA, mature miRNA, miRNA^*^ strands, hairpins loops, and pre-miRNA cleavage products in EVs (Chen et al., 2010; Melo et al., 2014). Over the course of 48 h, Melo et al. identified a sharp decrease in pre-miRNA abundance in previously purified exosomes, which coincided with a marked increase in corresponding mature miRNA levels. In addition, Melo et al. identified a role of the sialoglycoprotein CD43 in recruiting Dicer to cancer cell exosomes. Indeed, co-immunoprecipiation revealed an interaction between the two proteins and silencing of CD43 severely compromised the recruitment of Dicer to exosomes. Moreover, inhibiting Dicer activity in breast cancer exosomes significantly impaired growth in recipient malignant cells, providing evidence of its involvement in tumor progression.

Villarroya-Beltri et al. provided robust evidence of a sequence-specific mechanism involving the RBP hnRNPA2B1 in miRNA sorting to EVs (Villarroya-Beltri et al., 2013). The authors investigated activation-dependent changes in the miRNA repertoire of lymphoblasts and observed divergent trends in cells and EVs consistent with active, sequence-specific loading. Sequence alignments and targeted mutagenesis revealed a role of the GGAG motif in miRNA targeting to EVs. RNA pull-down experiments coupled to mass spectrometry identified three hnRNP factors specifically bound to EV-targeted miRNA. The author focused on hnRNPA2B1 and confirmed specific miRNA association by immunoprecipitation coupled to qPCR. They also showed that hnRNPA2B1 targeting to EVs is regulated by SUMO conjugation. Annexin A2 is a Ca^2+^-binding protein that contributes to link membrane-associated complexes to cytoskeletal components (Gerke et al., 2005). Annexin A2 exhibits sequence-specific RNA-binding activity and is involved in *c-myc* post-transcriptional regulation (Filipenko et al., 2004). Proteomic studies have revealed that Annexin A2 is among the most abundant proteins in EVs (Hagiwara et al., 2015). Hagiwara et al. provided evidence that Annexin A2 can bind miRNA in the presence of Ca^2+^ in diverse cancer cell lines (Hagiwara et al., 2015). The authors reported a global decrease in miRNA loading to cancer cell EVs upon Annexin A2 silencing.

Lipidomics studies based on mass spectrometry and nuclear magnetic resonance have unraveled profound differences in the composition of EV and plasma membranes (Choi et al., 2013). Lipid rafts are dynamic, detergent-resistant membrane microdomains enriched in sphingomyelin and depleted in phosphatidylcholine (de Gassart et al., 2003). Lipid rafts are overrepresented in EVs and have been involved in sorting proteins and RNAs to exosomes (de Gassart et al., 2003; Dubois et al., 2015). The sphingolipid ceramide is enriched in lipid rafts and implicated in membrane sorting during exosome budding (Megha and London, 2004; Trajkovic et al., 2008). Neutral sphingomyelinase (nSMase) is the rate-limiting enzyme in ceramide biogenesis and its inhibitor GW4869 has been used by several groups to restrict exosome release *in vitro* (Trajkovic et al., 2008; Yuyama et al., 2012; Essandoh et al., 2015). The “ceramide pathway” has emerged as an important route for miRNA loading to exosomes (Yuyama et al., 2012). Kosaka et al. provided evidence that metastatic cancer cells exert microenvironment remodeling of endothelial cells through exosome-associated miR-210 (Kosaka et al., 2013). This phenotype was abrogated by silencing nSMase in breast cancer cell lines, consistent with a role of the ceramide pathway in exosomal miRNA sorting.

Koppers-Lalic et al. reported that 3′ end uridylated miRNA isoforms are enriched in B cell exosomes, whereas 3′ end adenylated isoforms are poorly targeted and relatively enriched in cells (Koppers-Lalic et al., 2014). The authors extended their finding in EVs purified from human urine samples and concluded that non-templated terminal uridylation promotes miRNA sorting to EVs. Previous studies have shown that terminal adenylation increases transcript stability while uridylation has a destabilizing effect (Scott and Norbury, 2013), bridging the findings of Koppers-Lalic et al. to Batagov et al.'s conclusions that RNAs with short half-lives are enriched in EVs. Squadrito et al. investigated co-dependencies in miRNA and target mRNA levels in bone marrow-derived macrophages and corresponding EVs (Squadrito et al., 2014). The authors used IL-4 and genetic perturbations to alter the expression of miRNA and their target mRNA. Their observations suggest that the levels of endogenous target modulate miRNA sorting to EVs, likely through a relocation of RISC from P-bodies to MVEs. Indeed, several studies suggest that intracellular targeting of AGO2 complexes to endolysosomal compartments and exosomes reflects the dynamics of membrane-less organelles such as P-bodies and GW182-bodies (Siomi and Siomi, 2009).

## Recruitment of AGO2-bound miRNA to MVEs

Gibbings et al. investigated RISC subcellular distribution using cell fractionation, immunofluorescence and qPCR (Gibbings et al., 2009). They showed that “GW-bodies” containing AGO2 and its cofactor GW182 are distinct from canonical P-bodies and selectively congregate with MVEs. Immunogold labeling of monocoyte-derived exosomes revealed enrichments for GW182 but not DCP1A, a canonical P-body marker. Diverse miRNA and their target mRNA were enriched in the vicinity of GW182-positive MVEs. To understand how GW-182 bodies are recruited to MVEs, the authors silenced components of the endosomal sorting complexes required for transport (ESCRT), a highly conserved multisubunit machinery that localizes to MVEs and performs bending and scission of the membrane involved in protein and exosome release (Schmidt and Teis, 2012). They showed that depleting ESCRT components severely impairs miRNA silencing activity in the cell by monitoring let-7-a and miR-206 repression through reporter assays. Gibbings et al. thus established two key principles: (1) miRNA-loaded RISCs congregate at the site of exosome biogenesis and (2) ESCRT components regulate both exosome biogenesis and RNAi.

Independently of its involvement in exosome secretion, the ESCRT-II complex exhibits sequence-specific RNA-binding activity in metazoans. Irion et al. (Irion and St Johnston, 2007) focused on *bicoid* mRNA localization during *Drosophila* development. The study revealed that mutations in all three subunits of the ESCRT-II complex abolish the localization of *bicoid* mRNA at the anterior pole of the egg. The authors demonstrated a direct interaction between the N-terminal GLUE domain of VPS36 and stem-loop V in *bicoid* 3′UTR using UV-crosslinking and a yeast three-hybrid assay. They extended their finding in *Xenopus*, establishing conservation of the interaction in Vertebrates. ESCRT-II is thus at the crossroads of exosome biogenesis, RNAi and subcellular RNA localization, prompting speculations that the complex may contribute to sRNA sorting to exosomes. Kosaka et al. tested the hypothesis and depleted an ESCRT component, Alix, in HEK293 cells. In agreement with Gibbings et al. luciferase assays showed a reduction in intracellular silencing activity by miR-146. However, the amount of miR-146 in EVs was not altered by Alix depletion. EVs from Alix-depleted HEK293 cells contained miR-146 and silenced a reporter gene in recipient cells as efficiently as EVs released by untreated cells. Further efforts are required to elucidate the involvement of ESCRT components in sRNA sorting to EVs.

Recent work by McKenzie et al. provides an alternative mechanism of AGO2-miRNA relocation from P-bodies to MVEs (McKenzie et al., 2016). Echoing Cha et al.'s identification of KRAS signaling as a modulator of miRNA sorting to EVs, McKenzie et al. showed that KRAS-dependent activation of the MEK-ERK pathway inhibits AGO2 sorting to EVs. This work revisits a previously identified KRAS-dependent phosphorylation of serine residue 387 on AGO2 and demonstrates its implication in excluding AGO2-miRNA complexes from MVE association and exosome targeting. AGO2 targeting to exosomes thus reflects KRAS-MEK-ERK signaling status, which is impacted by environmental cues. These reconciliatory findings provide a possible explanation for discrepancies in previous reports regarding AGO2 levels in exosomes (Gibbings et al., 2009; Melo et al., 2014).

## Contrasting and “EV Sceptic” perspectives

We have reviewed examples of RNAi activity transfers across diverse species spanning the eukaryotic and prokaryotic domains of life. We then envisioned possible vehicles of sRNA transfer, including EVs, lipoproteins, soluble RNPs, and tunneling nanotubes. We emphasized emerging mechanisms of sRNA sorting to EVs in mammalian system, suggesting that these vesicles may contribute to cross-species sRNA transfers. EV association strongly enhances the stability of RNA molecules in the extracellular environment. In addition, examples of long-range transfers of biomolecules through EVs have been reported in diverse systems. In *C. elegans*, EVs transferred between worms contribute to the specification of male sexual behavior (Wang et al., 2014), while functional EV-associated transcripts encoding a Cre recombinase are shuttled across distant tumors in mice (Zomer et al., 2015). Transfers of EVs and delivery of molecular cargo from human to mouse cultured cells and from the protozoan pathogen *Trypanosoma cruzi* to human erythrocytes have been documented, suggesting that EVs can indeed serve as widespread mediators of interspecies RNA transfers (Valadi et al., 2007; Deolindo et al., 2013; Evans-Osses et al., 2015).

Numerous studies thus support the functionality of EV-associated sRNA populations in intercellular communication (Pegtel et al., 2010; Katsuda et al., 2014; Melo et al., 2014). However, contrasting reports resulting from careful quantitative assessments argue that EVs are poor vehicles for RNA transfers due to degradation upon recipient cell entry and/or insufficient cargo abundance. Kanada et al. examined the fate of nucleic acids contained in HEK293FT small exosome-like EVs and larger microvesicle-like EVs upon recipient cell entry (Kanada et al., 2015). They found that exosome-like EVs fail to transfer nucleic acids to murine 4TI recipient cells. Microvesicle-like EVs delivered reporter RNA, which was successfully amplified using a nested PCR approach 24 h after delivery. However, full-length and fragmented reporter RNA was undetectable 48 h after transfer assays, likely due to degradation in acidic lysosomal compartments. Plasmid-encoded Cre recombinase was efficiently loaded in microvesicle-like EVs as plasmidic (p)DNA, RNA and protein. Recombinase activity was stably transmitted to recipient cells, but exclusively through pDNA. These findings suggest that pDNA transfers may have confounded the conclusions of several studies using reporter constructions to investigate RNA transfers.

Chevillet et al. purified EVs from five human biological fluids and cell line media and used quantitative approaches to determine miRNA stoichiometric abundance in these samples (Chevillet et al., 2014). Regardless of the source, they found that EV samples contain low counts of individual miRNA, amounting at most to an average of (0.00825 ± 0.02) miRNA molecules per EV. While this result suggests that EVs are poor miRNA transfer vehicles *in vivo*, Chevillet et al. discussed diverse stoichiometric models to reconcile their assessments with reports of functional miRNA delivery. Indeed, population analyses are unable to determine the distributions of miRNA molecules in individual EVs. A low occupancy/high miRNA concentration model, wherein most EVs contain no miRNA molecule but a single EV carries several copies could be compatible with reports of functional transfers. Similarly, the kinetics of EV uptake could impact functionality in recipient cells, provided they internalize EVs at a high frequency. In the context of interspecies communication, it should be noted that organisms able to amplify systemic RNAi responses such as plants and nematodes may exhibit enhanced sensitivity to signals conveyed by a low number of initial miRNA copies.

Contrasting and skeptical reports are highly valuable to the EV field and should inform attentive methodological choices for future experiments. In transfer assays, reporter genes expressed from chromosomal insertions should be favored rather than plasmid-based approaches, effectively ruling out pDNA transfers. DNAse treatments should be used when specifically investigating the roles of EV-associated RNA. Imaging EV transfers through time-lapse analysis of high-resolution microscopy data could illuminate the kinetics of internalization. In addition, the fate of transferred biomolecules should be considered over the course of several days, documenting the association of transferred material with subcellular compartments of recipient cells that may impact stability. Dutiful application of these principles will reveal whether membrane vesicles can indeed spread silencing instructions across phylogenetic boundaries.

## Author contributions

FL wrote the manuscript and assembled the figures. EL provided oversight and revised the manuscript.

## Funding

EV research in the Lécuyer lab has been supported by the Cancer Research Society of Canada (grant #20227).

### Conflict of interest statement

The authors declare that the research was conducted in the absence of any commercial or financial relationships that could be construed as a potential conflict of interest.
